# Development and validation of a Vero cell DNA standard for residual DNA measurement suitable for quantitative PCR method

**DOI:** 10.3389/fmicb.2026.1708044

**Published:** 2026-05-14

**Authors:** Danhua Zhao, Yunpeng Wang, Xiaohong Wu, Leitai Shi, Jia Li, Yuhua Li, Shouchun Cao

**Affiliations:** 1 Department of Arbovirus Vaccines, National Institutes for Food and Drug Control, Beijing, China; 2 Research Units of Innovative Vaccine Quality Evaluation and Standardization, Chinese Academy of Medical Sciences, Beijing, China

**Keywords:** DNA residue, qPCR method, quantitative standard, standardization, Vero cells

## Abstract

**Introduction:**

The fluorescence quantitative PCR method for the detection of Vero cell residual DNA in human rabies vaccine urgently needs a national standard for Vero cell DNA quantification.

**Methods:**

Briefly, the genomic DNA of Vero cells was extracted, diluted to 100 μg/ml, and divided into 0.1-ml aliquots in sterile screw cap tubes. After DNA preparation, concentration calibration, applicability, and stability verification were carried out by multiple laboratories.

**Results:**

The concentration of the national standard for Vero cell DNA quantification (limited qPCR method) determined from this collaborative study was 107 ± 6 μg/mL, and the DNA purity (OD_260_/OD_280_) was 1.83. This standard is only suitable for the qPCR method, which has an average amplification efficiency of 92%. After accelerated degradation at 37 °C from 1 to 4 weeks, the standard showed amplification curves similar to those stored at −70 °C, with no significant difference in amplification efficiency, showing good stability.

**Conclusion:**

This study successfully developed a national standard for Vero cell DNA quantification using the quantitative fluorescence PCR method, which has significant application potential for standardizing Vero cell DNA residue detection in human rabies vaccines.

## 1 Introduction

Vero cells, formally known as African green monkey kidney cells, are a type of continuously growing cells. Yasumura and Kawakita, two scientists from Chiba University in Japan, obtained the cell line in 1962 during the primary culture of African green monkey kidney epithelial cells (Yasumura and Kawakita, 1963). Vero cells are easy to culture, suitable for mass production, and can be passaged continuously *in vitro*. Therefore, it has become one of the cellular substrates recommended by the World Health Organization (WHO) for human vaccines, such as the freeze-dried human rabies vaccine and freeze-dried inactivated Japanese encephalitis vaccine. As a continuous cell line, the DNA of Vero cells has tumorigenic risk. Therefore, the quality control of Vero cell residual DNA in vaccines is necessary (World Health Organization [WHO], 2014).

Drug regulatory agencies in various countries have formulated corresponding standards for Vero cell residual DNA in viral vaccines. Currently, there are three standards worldwide: no higher than 10 ng/dose, 100 pg/dose, and 10 pg/dose (World Health Organization [WHO], 2013; Pharmacopoeia Commission of the People’s Republic of China, 2020). According to the Pharmacopoeia of the People’s Republic of China (2010 Edition, Part III), the host cell residual DNA must be evaluated in the human rabies vaccine (Vero cells), and the limit is 100 pg/dose (Pharmacopoeia Commission of the People’s Republic of China, 2010). Therefore, the techniques to detect such a small amount of DNA residue must be extremely sensitive and stable. There are three main approaches for quantifying DNA residues: molecular hybridization techniques, DNA binding protein-based methods (Threshold^®^ immunoassay), and fluorescence quantitative PCR.

Fluorescence quantitative PCR technology refers to the method of adding fluorophores to the PCR reaction system, using the accumulation of fluorescence signals to monitor the entire PCR process in real-time, and quantitative analysis of unknown templates through standard curves, which can accurately quantify nucleic acid content, and has the advantages of high sensitivity, strong specificity, short time and high throughput detection. According to references (Cao et al., 2011; Vernay et al., 2019), the National Institutes for Food and Drug Control (China) developed the Taqman fluorescence quantitative PCR detection method based on the 172-bp target gene sequence unique to Vero cells (Zhao et al., 2025). The PCR amplification fragment length of this method is 154 bp, with a high sensitivity of up to 0.003 pg/10 μL. Using this method, there was no cross-reaction with the DNA of human diploid cells, Chinese Hamster Ovary (CHO) cells, yeast cells, and *Escherichia coli*. Through the ultrasonic fragmentation of DNA, the fragments between 100 and 2,000 bp has no effect on the detection. The results showed that this method was suitable for detecting Vero cell DNA residue in human rabies vaccines, especially in drug substance.

To further standardize this qPCR method for detecting Vero cell DNA residue, it is urgent to develop corresponding national standards. According to the requirements for the preparation of reference material in the Pharmacopoeia of the People’s Republic of China (2010 Edition, Part III), we successfully developed the national reference material for Vero cell DNA quantification applicable to the probe hybridization method (Cao et al., 2013), which was approved for use in 2012 (identification number: 250015–201101). The freeze-dried standard product contained protective agents, such as bovine serum albumin, and the load is 0.5 ml/tube. Considering the high sensitivity of the qPCR method and the precision requirement for freeze-dried product packaging, referring to the preparation schemes for CHO and *E. coli DNA* standards (Zheng et al., 2019; Wang et al., 2013), this national standard for qPCR Vero cell DNA quantification was prepared in liquid dosage form, and a flat-bottom screw cap centrifuge tube with low adsorption capacity was used for packaging (World Health Organization [WHO], 2021a).

## 2 Materials and methods

### 2.1 Materials

The Vero cell line was purchased from American Type Culture Collection (ATCC). Cells were cultured in Eagle’s minimum essential medium (Gibco, Waltham, MA) supplemented with 10% fetal bovine serum (FBS, Gibco) and 5% CO_2_ at 37°C and passaged every 2–4 days.

Genomic DNA was extracted from fresh cell cultures using the Blood & Cell Culture DNA maxi kit (Qiagen, Hilden, Germany).

The primers and probe information was as follows:

Forward primer: 5′-GCTTTCTGAGAAACTGCTCTGTGT-3′

Reverse primer: 5′-GGAAGATATTTCCTTTTTCACCATA GC-3′

Probe: 5′-CCTTCAAGAAGCCTTTCGCTAAG-3

### 2.2 Preparation and packaging of the Vero cell DNA standard

Due to the high demand for Vero cell DNA standard and the need for large-scale cell culture, Liaoning Chengda Biotechnology Co., Ltd., which has a reliable source of Vero cells, performed the large-scale culture of Vero cells. The Huzhou R&D Center for Nutrition and Health, Shanghai Institute of Nutrition and Health, and the Chinese Academy of Sciences were commissioned to extract DNA from the cultured Vero cells. The concentration and purity of the extracted DNA were evaluated and adjusted through the UV absorbance method to a concentration of 100 μg/mL and a purity between 1.8 and 2.0 (absorbance ratio at 260 and 280 nm). According to the target packing concentration of 100 μg/ml, 1-ml flat-bottom screw cap centrifuge tubes with low adsorption capacity were used for packaging. The product load was 100 μl/piece, and approximately 6,000 pieces were produced.

Packaging precision was monitored during its initial, middle, and final stages. According to the provisions of the Operating Procedures for the Stability and Uniformity Evaluation of Reference Materials for Biological Products (SOP No. NIFDC-SOP-S-5104), when the total unit number is beyond 500, the number of sampling units should be not less than 25; when the total number of units *N* is above 1,000, the number of sampling units could be 3 (symbol). The target packaging quantity in this study was 6,000; thus, the theoretical sampling quantity is 55. Herein, 30 tubes were sampled in each of the initial, middle, and final stages of packaging, so a total of 90 samples were selected to evaluate the packaging accuracy. The weighing method was adopted for testing, and the weight deviation was calculated as follows: (actual weight − average weight)/average weight × 100.

### 2.3 Collaborative calibration and determination of Vero cell DNA standard concentrations

Considering the organizations using this standard product, eight laboratories qualified for quantitative nucleic acid detection and vaccine lots release were selected for collaborative calibration.

The concentration and purity of the Vero cell DNA were determined using the UV absorbance method, and the optical densities at 260 and 280 nm were recorded. Three parallel independent tests are required for each collaborative laboratory; the average value of three tests was regarded as the final test result. The candidate standards were uniformly tested after 2-fold dilution with a DNA diluent (TE buffer) to ensure the test accuracy and minimize the inter-laboratory operating errors.

After statistical treatment of 24 groups of data from eight laboratories, the concentration of the Vero cell DNA standards was determined based on the arithmetic mean value.

### 2.4 Applicability verification of the Vero cell DNA standards

The essential criterion for a qPCR standard is that it should not contain inhibitors. The purity of the standard prepared in this study met the requirements. TE buffer was selected as the solvent of this standard, which did not inhibit the PCR test; hence, this Vero cell DNA standard still had a typical amplification curve after dilution. Therefore, it can be preliminarily concluded that the prepared standard is suitable for fluorescence quantitative PCR detection. To further verify the suitability of the standard, verification was extended to a total of 15 qualified test institutions.

Briefly, 4 μL of the Vero cell DNA standard was added into 140 μL of the DNA diluent, labeled as ST0, and then immediately centrifuged for 10 s after mixing, repeating thrice to ensure thorough mixing. ST1–ST7 were made from a series of 10-fold gradient dilutions (Table 1), and ST2–ST7 were selected for fluorescence quantitative PCR detection.

**TABLE 1:** Dilution of the standard.

Dilution tube	Dilution volume	Concentration
ST0	4 μL Vero DNA standard + 140 μL DNA diluent	3 ng/μL
ST1	10 μL ST0 + 90 μL DNA diluent	300 pg/μL
ST2	10 μL ST1 + 90 μL DNA diluent	30 pg/μL
ST3	10 μL ST2 + 90 μL DNA diluent	3 pg/μL
ST4	10 μL ST3 + 90 μL DNA diluent	300 fg/μL
ST5	10 μL ST4 + 90 μL DNA diluent	30 fg/μL
ST6	10 μL ST5 + 90 μL DNA diluent	3 fg/μL
ST7	10 μL ST6 + 90 μL DNA diluent	0.3 fg/μL

### 2.5 Stability evaluation of the Vero cell DNA standards

Vero cell DNA is theoretically stable. In addition, the DNA extraction and packaging process employed in this study involved being kept on the ice and in the biosafety cabinet for aseptic operation to minimize external contamination and keep the stability of DNA. For a preliminary investigation on the stability of the standard, the standard was placed at 37 °C for 1, 2, 3, 4, and 8 weeks according to the requirements of the Pharmacopoeia of the People’s Republic of China. Then, the stability of the standards stored for different periods was compared with that stored at −70 °C (0 weeks) via agarose gel electrophoresis and fluorescence quantitative PCR. The results of agarose gel electrophoresis can reveal the degradation of DNA directly by observing the electrophoretic band shape of DNA. The stability of the standard can also be investigated by evaluating the change in amplification efficiency during fluorescence quantitative PCR when the standard is diluted.

### 2.6 Data analysis

#### 2.6.1 Calculation of the amplification efficiency

E = (10^–1/slope^ − 1) × 100%.

The slope is derived from the standard curve by plotting Ct values against the logarithm of the initial template quantity.

#### 2.6.2 Calculation of sample coefficient of variation (CV)

CV = Standard deviation of detection value/mean detection value × 100%.

#### 2.6.3 Statistical analysis

The CV was calculated for all experimental data using Microsoft Excel (Microsoft Corporation, Redmond, WA, United States)

## 3 Results

### 3.1 Preparation and packaging of Vero cell DNA standards

The mean measured concentration of the standard (batch No. Y20190311) was 112.6 μg/mL, and the purity was 1.85 (Table 2). After dilution according to the target packaging concentration of 100 μg/mL (batch no. Y20190311, diluted), the mean measured concentration and purity were 99.7 μg/mL and 1.88, respectively (Table 3). The final load is 100 μL/piece, and 5,066 packages were manufactured.

**TABLE 2:** Concentration and purity of Vero cell DNA candidate standard stock solutions.

Group	Trial	OD_260_	OD_280_	DNA concentration (μg/mL)	DNA purity OD_260_/OD_280_
I	1	2.279	1.245	113.9	1.83
	2	2.212	1.214	110.6	1.82
	3	2.298	1.249	114.9	1.84
II	1	2.220	1.181	110.7	1.88
	2	2.246	1.195	112.2	1.88
	3	2.267	1.212	113.4	1.87
Mean	/	/	/	112.6	1.85

**TABLE 3:** Concentration and purity of the diluted Vero cell DNA candidate standard solutions.

Group	Trial	OD_260_	OD_280_	DNA concentration (μg/mL)	DNA purity OD_260_/OD_280_
I	1	2.004	1.064	100.2	1.88
	2	1.992	1.060	100.0	1.88
	3	1.958	1.047	98.8	1.87
Mean	/	/	/	99.7	1.88

Packaging precision evaluation showed that the accuracy in the initial packaging stage was ± 5%, ± 4% in the middle stage, and between −6 and 5% in the final stage (Table 4). Although the packaging accuracy did not meet the requirements of the Pharmacopoeia of the People’s Republic of China, it has no impact on the subsequent application of the standard product. Because this standard is indicated as a liquid dosage, and its concentration will not be affected by the accuracy of packaging. Moreover, only 4–5 μL is needed for each test.

**TABLE 4:** Test results of the Vero cell DNA standard packaging accuracy evaluation.

Initial stage				Middle stage				Final stage			
Number	Load uniformity (%)	Number	Load uniformity (%)	Number	Load uniformity (%)	Number	Load uniformity (%)	Number	Load uniformity (%)	Number	Load uniformity (%)
1	4.0	16	0.4	31	1.8	46	−1.0	61	−1.6	76	−0.1
2	−1.9	17	−0.1	32	−3.9	47	−0.7	62	−2.7	77	−5.4
3	−1.4	18	−4.9	33	0.9	48	−0.7	63	1.9	78	−2.8
4	3.1	19	−0.1	34	0.5	49	0.7	64	−0.9	79	0.2
5	−0.5	20	4.4	35	4.4	50	−2.1	65	−0.5	80	−3.0
6	3.1	21	0.6	36	1.4	51	1.0	66	0.6	81	0.1
7	−1.9	22	−0.9	37	−1.9	52	−0.8	67	−3.9	82	−3.8
8	0.4	23	−1.8	38	−2.2	53	−0.5	68	−2.4	83	4.5
9	1.0	24	−0.8	39	0.4	54	−1.5	69	−1.6	84	−2.8
10	1.0	25	−2.9	40	0.8	55	0.6	70	0.4	85	−1.9
11	1.0	26	2.5	41	−0.8	56	0.4	71	1.3	86	2.9
12	1.5	27	0.3	42	−0.8	57	0.9	72	−2.0	87	−3.4
13	3.3	28	−4.7	43	2.4	58	1.3	73	−5.6	88	1.0
14	1.8	29	−0.7	44	−0.7	59	1.1	74	−5.7	89	2.3
15	1.1	30	5.3	45	−1.9	60	0.2	75	−0.8	90	0.0
−4.9%∼5.3%				−3.9%∼4.4%				−5.6%∼4.5%			

### 3.2 Collaborative calibration of Vero cell DNA standard

**FIGURE 1:**
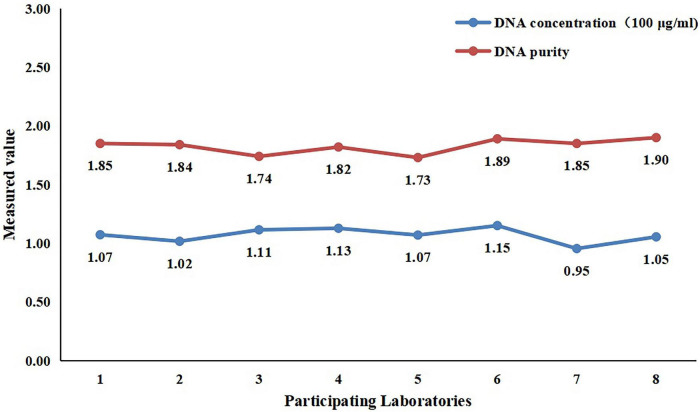
Results of the Vero cell DNA standard concentration and purity obtained during the collaborative study.

All eight laboratories involved in the collaborative calibration study submitted data as required. A total of 24 tests were performed, all of which produced valid data (Table 5). After statistical analysis, the detection CV value among various laboratories was 5.9%, the mean concentration value obtained from the 24 tests was 107 ± 6 μg/mL, and the DNA purity (OD_260_/OD_280_) was 1.83, which met the requirements (Figure 1).

**TABLE 5:** Summary of the collaborative study results of Vero cell DNA standard concentration.

Collaborative laboratory	Trial	OD_260_	OD_280_	Dilution factor	DNA concentration (μg/mL)	DNA purity OD_260_/OD_280_
01	1	1.116	0.603	2	111.6	1.85
	2	1.038	0.561		103.8	1.85
	3	1.067	0.573		106.1	1.85
02	1	1.014	0.551	2	101.4	1.84
	2	1.017	0.556		101.7	1.83
	3	1.018	0.553		101.8	1.84
03	1	1.112	0.643	2	111.2	1.73
	2	1.109	0.639		110.9	1.74
	3	1.121	0.644		112.1	1.74
04	1	1.124	0.618	2	112.4	1.82
	2	1.131	0.621		113.1	1.82
	3	1.126	0.619		112.6	1.82
05	1	1.231	0.763	2	123.1	1.61
	2	1.007	0.564		100.7	1.79
	3	0.968	0.543		96.8	1.78
06	1	1.118	0.592	2	111.8	1.89
	2	1.149	0.619		114.9	1.86
	3	1.182	0.614		118.2	1.93
07	1	0.856	0.436	2	85.6	1.96
	2	0.983	0.549		98.3	1.79
	3	1.024	0.567		102.4	1.81
08	1	1.053	0.553	2	106.0	1.90
	2	1.059	0.560		106.0	1.89
	3	1.049	0.552		104.0	1.90
Mean	/	/	/	/	107	1.83

### 3.3 Applicability validation of the Vero cell DNA standard by qPCR method

The verification results of 15 laboratories showed that the manufactured standard had typical amplification curves after 10-fold gradient dilution, and all parameters of its fitting curve were compliant. The R^2^ values of the fitting curve were all above 0.990, with an average of 0.998. The mean C_*T*_ values of the standard series (ST2 to ST7) were as follows: 12.297 at 30 pg/μL (ST2), 15.718 at 3 pg/μL (ST3), 19.316 at 300 fg/μL (ST4), 22.815 at 30 fg/μL (ST5), 26.337 at 3 fg/μL (ST6), and 29.721 at 0.3 fg/μL (ST7). The assay demonstrated good linearity across the tested concentration range, with an average amplification efficiency of 93% (Table 6). The collaborative experimental results from multiple laboratories indicate that the standard prepared was suitable for fluorescence quantitative PCR detection.

**TABLE 6:** Applicability verification results of Vero cell DNA candidate standard for fluorescent quantitative PCR test.

Laboratory number	C_*T*_ value of ST2	C_*T*_ value of ST3	C_*T*_ value of ST4	C_*T*_ value of ST5	C_*T*_ value of ST6	C_*T*_ value of ST7	R^2^	Slope	Amplification efficiency (%)
01	12.220	15.730	19.420	22.900	26.470	29.880	1.000	−3.540	92
02	12.666	15.972	19.643	23.253	26.276	29.964	0.998	−3.458	95
03	11.790	15.267	18.691	22.257	26.614	29.345	0.996	−3.582	90
04	11.689	15.045	18.592	22.077	25.691	29.462	0.999	−3.551	91
05	12.946	16.439	19.824	23.380	26.563	30.637	0.995	−3.497	93
06	11.730	15.056	18.567	22.078	25.626	28.648	0.998	−3.423	96
07	11.669	15.453	18.986	22.464	25.954	29.608	0.998	−3.562	91
08	13.760	17.010	20.750	24.060	27.480	30.460	0.999	−3.377	98
09	12.083	15.387	19.105	22.868	26.386	29.713	0.997	−3.569	91
10	10.778	14.290	18.056	21.503	24.740	28.722	0.999	−3.558	91
11	13.802	16.948	20.384	23.830	27.167	30.465	0.999	−3.355	99
12	11.324	15.061	18.815	22.243	26.161	29.932	0.999	−3.710	86
13	12.295	15.449	19.210	22.634	26.112	29.468	0.999	−3.465	94
14	11.866	15.450	19.123	22.474	26.447	28.945	0.998	−3.487	94
15	13.830	17.210	20.580	24.210	27.370	30.560	0.999	−3.300	101
Mean	12.297	15.718	19.316	22.815	26.337	29.721	0.998	−3.496	93
CV value (%)	7.575	5.345	4.078	3.468	2.671	2.167	0.132	/	4.161

### 3.4 Stability evaluation of the Vero cell DNA standard

**FIGURE 2:**
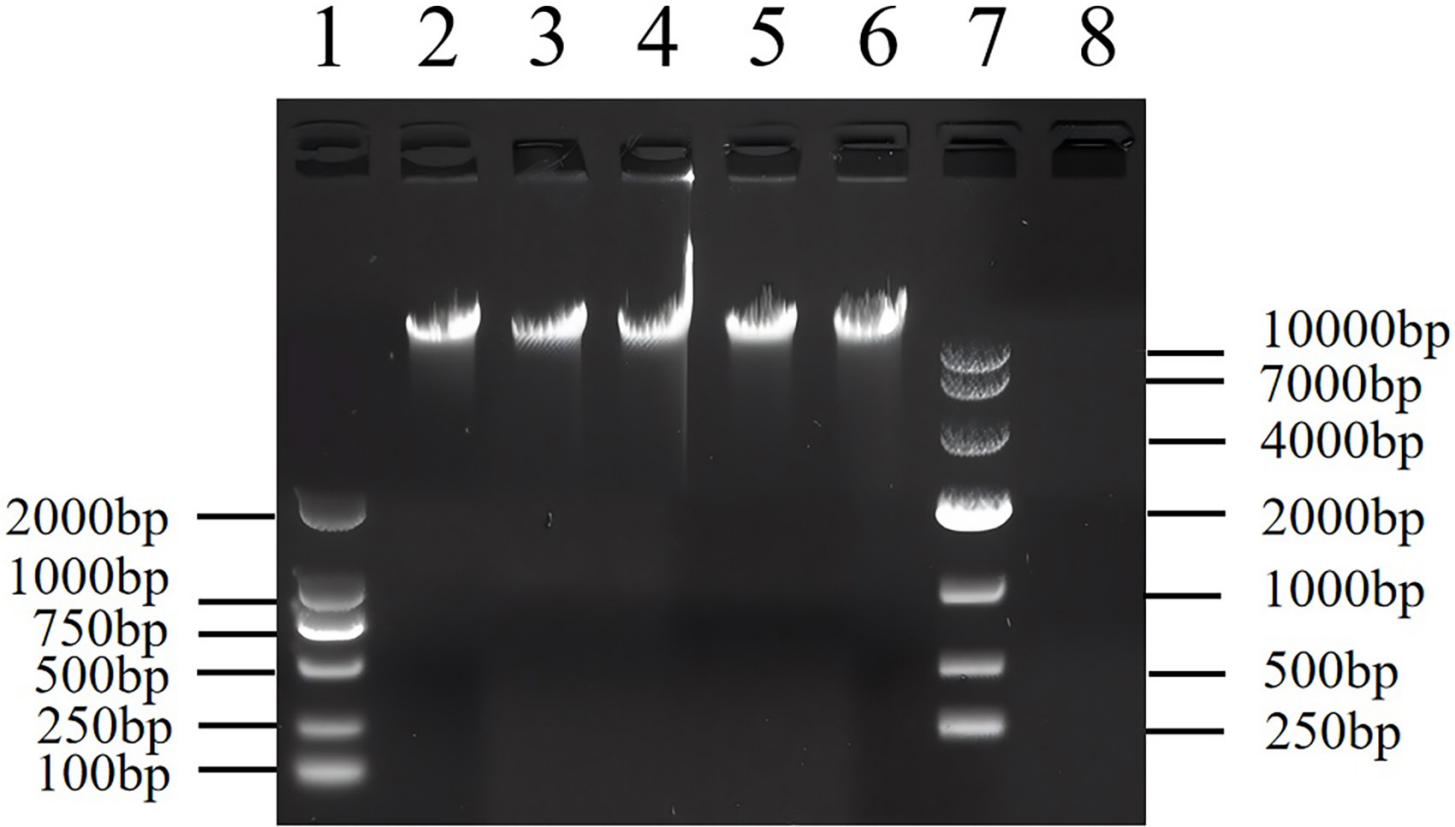
Results of the 0.8% agarose gel electrophoresis of the DNA standards after accelerated stability testing at 37°C for various durations. Lane 1 is a DNA marker, Lane 2 is a control sample (0 week), lane 3 is a 1 week-treated sample, lane 4 is a 2 week -treated sample, lane 5 is a 3 week-treated sample, lane 6 is a 4 week-treated sample, lane 7 is another DNA marker and lane 8 is a no-template control.

**FIGURE 3:**
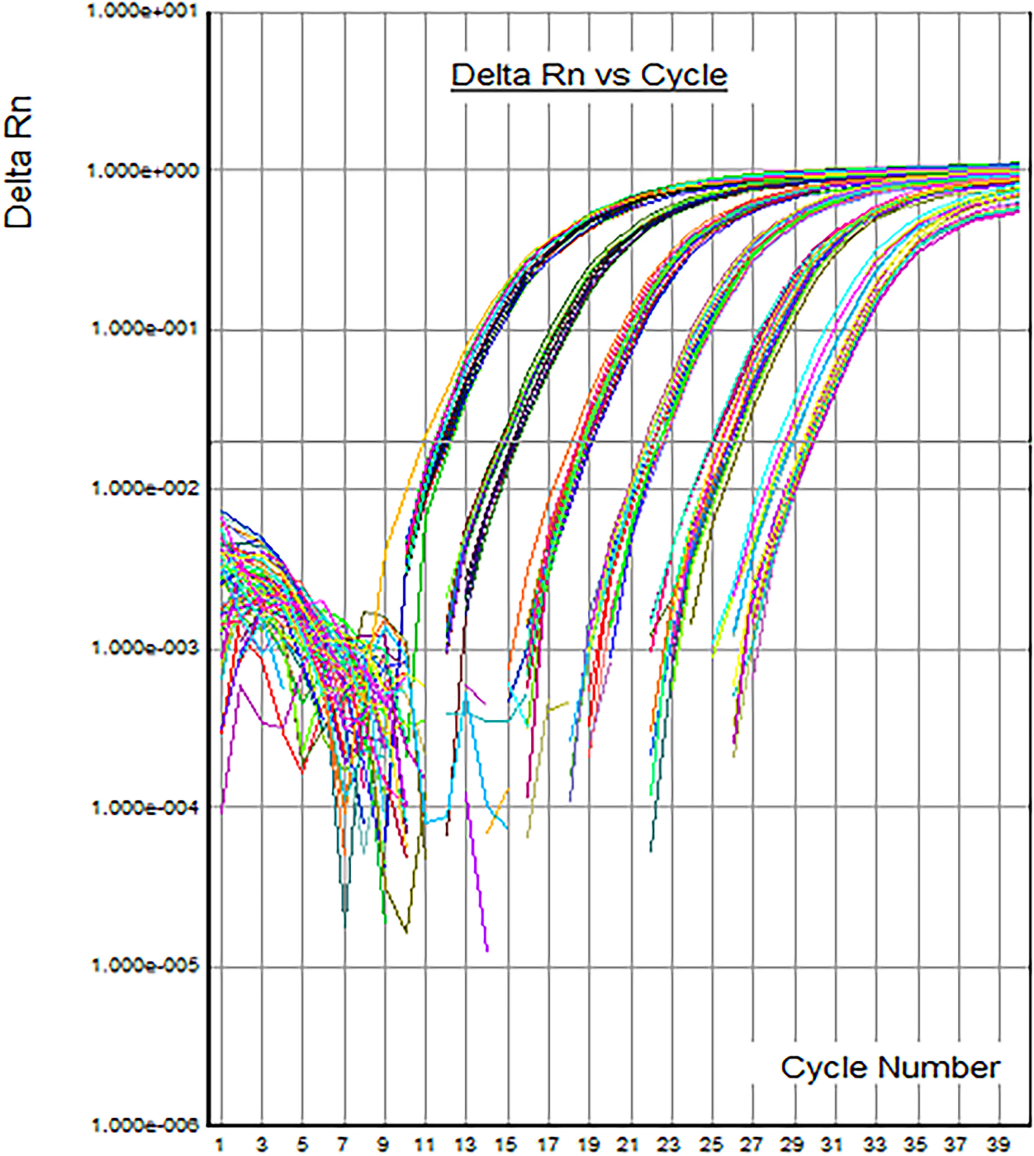
Fluorescence quantitative PCR amplification curve of the diluted DNA standards after accelerated stability testing. Typical amplification curves were observed after 0, 1, 2, 3, and 4 weeks of accelerated stability testing at 37 °C.

DNA standards incubated at 37 °C for different periods (i.e., 0, 1, 2, 3, 4 weeks) were subjected to 0.8% agarose gel electrophoresis (Figure 2). The size of electrophoretic bands did not differ after different durations of accelerated stability testing at 37 °C, and had no significant difference from that under normal storage conditions, directly indicating that the candidate DNA standard does not degrade under the tested conditions. The results of fluorescence quantitative PCR showed that typical amplification curves were observed (Figure 3) after 0, 1, 2, 3, and 4 weeks of accelerated stability testing at 37 °C. The amplification efficiency ranged from 89 to 94%, the CV value was 2.3%, and there was no significant difference in amplification efficiency (Table 7). Hence, the Vero cell DNA standard prepared in this study is stable and can meet the requirements of fluorescence quantitative PCR detection.

**TABLE 7:** qPCR detection results of the Vero cell DNA standard after thermal accelerated degradation.

Standing time at 37 °C	C_*T*_ value of ST2	C_*T*_ value of ST3	C_*T*_ value of ST4	C_*T*_ value of ST5	C_*T*_ value of ST6	C_*T*_ value of ST7	R^2^	Slope	Amplification efficiency (%)
0 week	12.087	15.314	18.962	22.394	25.830	29.508	0.998	−3.488	94
1 week	11.698	15.066	18.864	22.237	25.798	29.609	0.998	−3.575	90
2 weeks	11.684	14.980	18.797	22.195	25.883	29.760	0.999	−3.614	89
3 weeks	11.375	14.599	18.452	21.842	25.318	28.885	0.999	−3.517	92
4 weeks	11.257	14.705	18.330	21.825	25.171	28.605	0.998	−3.475	94
Mean	11.620	14.933	18.681	22.099	25.600	29.273	0.998	−3.534	92
CV value (%)	2.789	1.919	1.470	1.146	1.289	1.710	0.055	/	2.479

## 4 Discussion

Vero cells are one of the cellular substrates recommended by WHO for vaccine production. They can be continuously passaged and cultured on a large scale, especially with the application of microcarrier technology. Approximately 80% of the cellular substrates for viral vaccine production currently comprise Vero cells, especially in developing countries, such as the human rabies vaccine in China. Human rabies vaccines produced from Vero cells account for more than 90% of the rabies vaccine market in China, which is used by approximately 10 million people annually. The host DNA of continuous cell lines has tumorigenic potential, and reports from the WHO claim that it is safe within 150 generations (World Health Organization [WHO], 2013; Sheng-Fowler et al., 2009). Therefore, it is essential to exercise strict control over DNA residues in the final vaccine products to ensure their safety. Due to the high demand for vaccines and the expansion of the target population to infants and young children, the Chinese drug regulatory authorities attach significant importance to the risk assessment of their tumorigenic potential, from quality standards to detection and inspection methods. The method included in the 2015 edition of the Pharmacopoeia of the People’s Republic of China for detecting exogenous DNA residues in Vero cell products is the “probe hybridization method” (3407). The advantage of this method is that it is easy and convenient to perform, and it has been used in domestic and foreign production enterprises. However, the detection results of this method involve semi-quantitative analysis, which is easily affected by subjective judgment. In addition, its operation time is long, and the procedures are complicated, which can affect the hybridization results, inducing poor reproducibility and other issues.

Fluorescent quantitative PCR is based on PCR amplification. Using a pair of primers and a specific fluorescent probe, sequence amplification can be detected using the fluorescence signal, which can be monitored in real-time to conduct a quantitative analysis of the template concentration in the sample (André et al., 2016; World Health Organization [WHO], 2021b). Quantitative PCR can reflect the concentration of the product in real-time, wherein a standard curve can be drawn based on the data generated using standards with a known concentration. Then, the concentration of the initial template can then be calculated from the standard curve generated. The fluorescent quantitative PCR method has the advantages of quantifiability, high sensitivity, and high throughput, which is especially suitable for process control and monitoring before and after vaccine purification during vaccine production. The National Institutes for Food and Drug Control (China) has established a Taqman fluorescence quantitative PCR detection method based on the 172-bp target gene sequence unique to Vero cells (Cao et al., 2011; Vernay et al., 2019), which produces a PCR amplicon 154 bp in length; thus, this primer-probe set is labeled as “the 154 primer-probe.” This sequence can be detected with a high sensitivity, up to 0.003 pg/10 μl. It has no cross-reaction with the DNA of human diploid cells, CHO cells, yeast cells, and *E. coli*. After ultrasonic DNA fragmentation, fragments with lengths between 100 and 2,000 bp do not affect the detection efficiency (André et al., 2016). Test results showed that this method was suitable for detecting DNA residues in Vero cells of human rabies vaccines, especially in the drug substance. Although this method is a conventional technique, the lack of Vero cell DNA standard for detection hinders the standardization of this method. Hence, the development and standardization of host DNA residue measurement methods has become an urgent technical problem in quality supervision and inspection.

As a quantitative standard, the DNA concentration was first quantified by corroborating the data provided by eight qualified independent laboratories to ensure the accuracy of the quantification. After collaborative calibration and statistical analysis, the arithmetic average was taken as the final DNA concentration (107 ± 6 μg/mL; purity (OD_260_/OD_280_), 1.83). Next, the packaging accuracy was evaluated during the initial, middle, and final stages of production. The analysis results showed that the accuracy of the whole packaging process was within ± 6%. Although this result is not within the requirements of the Pharmacopoeia of the People’s Republic of China, it has no impact on the subsequent application of the standard product. Because this standard will be used as a liquid dosage, and its concentration will not affected by the accuracy of packaging. Moreover, only 4–5 μL will be needed for each test.

The fluorescence quantitative PCR method can accurately quantify nucleic acids with high sensitivity and strong specificity in a short time at high throughput, among other advantages. The latest draft of the United States Pharmacopoeia only included the qPCR method. In this study, 15 qualified laboratories in China conducted fluorescence quantitative PCR tests on the manufactured standard. All of them observed typical amplification curves, the parameters of which met the requirements, indicating that this standard is indeed suitable for qPCR tests (World Health Organization [WHO], 2021b; European Directorate for the Quality of Medicines, 2023). The successful development of this standard lays the foundation for the standardization of qPCR quantification of host DNA residues for Vero cells. Furthermore, this standard has been officially approved in China (identification number: 250021–201901), which has important theoretical significance and practical value for vaccine manufacturers and quality inspectors.

## Data Availability

The original contributions presented in this study are included in the article/supplementary material, further inquiries can be directed to the corresponding authors.
